# An AI Safety Monitoring System for Electric Scooters Based on the Number of Riders and Road Types

**DOI:** 10.3390/s23229181

**Published:** 2023-11-14

**Authors:** Woo-Jin Jang, Dong-Hyun Kim, Si-Hyung Lim

**Affiliations:** 1 Department of Mechanical Systems Engineering, Graduate School, Kookmin University, Seoul 02707, Republic of Korea; pkqoxmf@naver.com; 2 CRIG Corporation, Changwon 51522, Republic of Korea; dhkim@crig.co.kr; 3 School of Mechanical Engineering, Kookmin University, Seoul 02707, Republic of Korea

**Keywords:** accelerometer, artificial intelligence, conductive film, electric scooter

## Abstract

Electric scooters are quickly becoming a popular form of mobility in many cities around the world, which has led to a surge in safety incidents. Moreover, electric scooters are not equipped with safety devices for riders, which can lead to serious accidents. In this study, a footrest, data-collection module, and accelerometer module for electric scooters were developed to prevent various accidents caused by the rapid increase in the use of electric scooters. In the experiment, the boarding data of the electric-scooter riders were collected from the footrest and data-collection module. Moreover, the driving data of the electric scooters for different road types were collected with the accelerometer module. We then trained two artificial intelligence (AI) models based on convolutional neural networks (CNNs) for different types of data. When we considered the learning accuracy and mean square error (MSE), which are performance indicators of the ability of the trained AI models to discriminate data, for each AI model, the learning accuracy converged to 100% and the MSE converged to 0. Further, this study is expected to help reduce the accident rate of electric scooters by resolving the causes of frequent accidents involving electric scooters around the world.

## 1. Introduction

An electric scooter (popularly known as electric scooter) is a mobility device operated by one person, similar to a bicycle or motorcycle, and is widely used regardless of the gender and age of the driver owing to its higher accessibility compared to other mobility devices that require expertise, including certification. In particular, S. Gossling has argued that micro mobility, such as electric scooters, can be a radical transportation innovation with the potential to challenge existing transportation systems by redistributing transportation infrastructure in dense urban centers [1]. Thus, these factors promoted electric scooters as the most popular form of a mobility device today, thereby contributing significantly to the expansion of urban transportation systems worldwide.

With the recent interest in electric scooters and development of convenient services that allow users to rent them through simple procedures, the usage rate of electric scooters has increased rapidly. Even with the rapidly increasing popularity of electric scooters, traffic laws related to them are yet to be established, resulting in the increase in accidents related to electric scooters globally. In addition, electric scooters do not have safety devices that ensure the safety of the rider, who is exposed to the environment. Therefore, in the event of an accident, both the victim and rider are seriously injured. According to K. Kazemzadeh et al., various accidents have occurred frequently due to insufficient consideration of traffic enforcement (wearing helmets, prohibition of drunk driving, etc.) for electric scooters, emphasizing the need for further research on the safety characteristics of electric scooters [2]. As such, accidents caused by electric scooters have emerged as a new global health problem, with no clear solution yet in any country.

Existing studies have analyzed the correlation between various factors to determine the severity of electric-scooter accidents. However, the lack of basic data on electric scooters has caused difficulty in analyzing various aspects of electric-scooter accidents, which have increased rapidly in a short period of time, and consequently, in solving this problem. Thus, several studies have been conducted to analyze basic data on electric scooters [3,4,5,6,7,8,9,10].

Since then, various studies have been conducted on the factors that contribute to the safety risks of electric scooters. As a result, various disciplines have analyzed the safety-unaware behaviors of drivers, such as driving under the influence of alcohol, not wearing a helmet, and illegal parking. In one of these studies, S. Carrese et al. proposed a solution to the growing problem of illegally parked electric scooters that do not comply with road traffic regulations using mathematical optimization modeling and algorithmic techniques, and proposed the effective deployment of professional personnel to manage illegally parked electric scooters [11].

Several reasons have contributed to the rapid increase in accidents involving electric scooters, of which the two most common factors are carrying more than the prescribed number of passengers and contact with pedestrians while driving on the sidewalk. T. Brauner et al. conducted an investigation into the main causes of motorized scooter accidents reported in the media in Germany and found that riding on sidewalks was the most common cause [12]. According to N. Haworth et al., riding on sidewalks (undefined roads) accounted for the highest number of accidents overall, and multiperson riding (passenger doubling) was found to be a factor in accidents involving riders using electric scooters through shared services [13]. First, for accidents caused by multiperson riding on electric scooters, electric scooters are designed to be a single-person mode of transportation. However, in its actual operation, it can be overloaded with more than the prescribed number of passengers. If the driver cannot properly control the center of the vehicle and needs to pass through a rough road or make a sharp turn on a corner, a rollover accident can occur, which can injure the passengers. In the second case, when the driver rides the electric scooter without distinguishing between the paved road and sidewalk, a collision accident can occur if the driver is not aware of a pedestrian or vehicle coming out of a blind spot due to the driver’s limited field of view.

Researchers have attempted to investigate the interior and exterior of an electric scooter to solve electric-scooter collision accidents caused by the aforementioned cases. E. Kim et al. developed an artificial intelligence (AI) system that can determine in real time whether the driver is concentrated on driving by collecting driving data according to the driving concentration of the electric-scooter driver [14]. However, a limitation of the developed AI system is its inability to distinguish the road being traveled. S. Seo attempted to prevent collisions between electric scooters and pedestrians by generating a noise with varying intensity based on the driving speed [15]. However, this device relies only on the power of the electric scooter and it cannot distinguish the number of riders. H. J. Park et al. developed a multifunctional helmet that can be worn by electric-scooter drivers and developed an AI system that can collect data on accident factors, such as not wearing a helmet and speeding, in real time [16]. However, this AI system relies on the data collected from the helmet, which makes it difficult to be used in shared scooter services with high helmet loss rates. Moreover, the response to multiperson boarding cannot be obtained because the data are collected based on a single person. M. S. Miah et al. developed an object-recognition AI model using surveillance cameras to detect illegal activities of electric-scooter riders, such as multiperson driving and not wearing helmets [17]. However, the developed AI model cannot identify sidewalks and is limited to areas installed with surveillance cameras.

In this study, we developed a module that can be attached to electric scooters to collect real-time data. Consequently, an AI model that determines the number of riders and sidewalk driving was generated to effectively prevent these factors from causing electric scooter accidents. Data collection was performed using an electric-scooter footrest, a data-collection module, and an accelerometer module. The accelerometer module has been actively utilized as a road-estimation method in several studies [18]. In this study, it was used to prevent pedestrian collisions caused by electric scooters driven on sidewalks. The footrest and data-collection module for electric scooters were used to address the problem of overloading during boarding. The footrest generates an electric signal based on the pressure applied to the footrest by the driver boarding the vehicle. Subsequently, the electric signal is received by the data-collection module. We used deep learning to build an AI model to determine the accident factors based on data measured with the data-collection and accelerometer modules, respectively, and evaluated the performance of the built AI model. As a result, the footrest, data-collection module, and accelerometer module developed in this study for electric scooters provided a solution to significantly reduce electric-scooter accidents by preventing frequent accident factors.

## 2. Materials and Methods

### 2.1. Module Set Up

**Figure 1:**
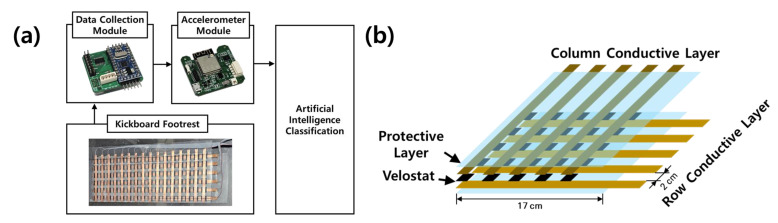
(**a**) Block diagram of the developed modules and AI model. (**b**) Schematic of the force-sensitive sensor array used as the footrest.

In this study, modules were produced for each experiment and data were collected for each of their functions to solve the two main causes of accidents when driving an electric scooter. The module operation is shown in the block diagram in Figure 1a.

To address the first case, which is overloading, a footrest and data-collection module were used. The footrest for the electric scooter was fabricated with an array of force-sensitive sensor layers consisting of 16 × 9 columns of Velostat, a force-sensitive material, according to the size of the commercially available electric scooter footrest to obtain the pressure information of the rider. The footrest was constructed with protective, conductive, and Velostat layers arranged sequentially to allow data collection with the Velostat. The schematic of the force-sensitive sensor array used for the footrest is shown in Figure 1b. The outermost protective layer consists of a lightweight polypropylene film, which does not conduct electricity nor affect the pressure measurements. The conductive layer is composed of a copper sheet that transmits electrical signals; each composed of rows and columns that are in contact to form a net-like structure. Finally, the Velostat layer is composed of Velostats cut into small square pieces and placed at the intersection of the conductive layers composed of rows and columns, achieving a total of 144 sensors that can identify the relative position of the Velostat on the footrest. The fabricated footrest sends an electrical signal to the data-collection module via wires attached to both ends of the conductive layer to determine the occupant’s position. This electrical signal changes in response to the varying resistance of the Velostat due to the pressure of the occupant. The data-collection module reads this electrical signal as an analog input and collects it.

The data-acquisition module consists of an analog multiplexer and an Arduino. The analog multiplexer scans the 16 × 6 sensor array per row to obtain the value of each sensor array point. Each element of the sensor array is classified as either high or low based on the generated pressure, which is binary, to achieve fast data processing and low power consumption. Finally, the Arduino switches the rows and columns to scan the value of each element. After digital signal processing, the value of each element is sent to the accelerometer module.

The accelerometer module was used to address the problem of collision between an electric scooter and a pedestrian or vehicle on sidewalks, which is the second cause of electric-scooter safety accidents. The accelerometer module is made of a printed circuit board that contains an accelerometer and a Wi-Fi wireless communication module. The accelerometer has a measuring range of ±16 g. In addition, the Wi-Fi wireless communication module capable of 2.4 GHz communication was used to wirelessly receive the data output from each sensor. The ESP-32s module used in this study supports Wi-Fi communication in the 2.4 GHz frequency band, supports network protocols, such as TCP/UDP, and supports a wide range of UART baud rates of up to 4,608,000 bps. Each sensor is connected to the Wi-Fi wireless communication module via I2C communication and transmits data to the terminal via UDP communication. The accelerometer module integrates the data measured with the accelerometer and the passenger boarding data received through serial communication and transmits them to the smartphone through the Wi-Fi wireless communication module.

### 2.2. Experimental Methods

Prior to the experiment, a threshold was set for the data-collection module to eliminate the pressure caused by fine wrinkles when the footrest is pressed. In particular, when a rider steps onto the electric scooter, the rider’s weight reduces the resistance of the Velostat attached to the coordinates corresponding to where the foot is placed, resulting in a high signal. The value measured is then transferred to each pin of the multiplexer through the analog-to-digital converter (ADC); only the coordinates measured using the output above the threshold are stored, whereas the rest of the coordinates are set to zero. Then, the boarding data transmitted to the accelerometer module are transmitted to the collection terminal (smartphone) using a wireless Wi-Fi communication through the built-in Wi-Fi function of the accelerometer module. In this process, the collection terminal (smartphone) receives the data from each module and transmits the boarding data to the unity-based self-developed smartphone software to obtain the output and store it on the screen. The boarding data stored in the developed software can be checked by connecting a separate PC for the analysis and a collection terminal (smartphone).

**Figure 2:**
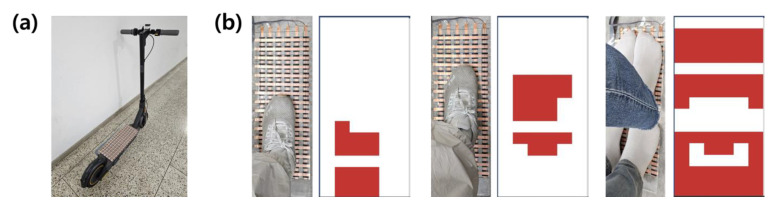
(**a**) Actual attachment of the electric-scooter footrest in the experiment. (**b**) Types of boarding data collected in the experiment and contour map measured from the footrest (one person in the back part, one person in the middle part, and two people on board).

The electric-scooter footrest and data-collection module are attached to the electric scooter as shown in Figure 2a. Data are collected through the experimental configuration, as shown in Figure 2b, to prevent overloading problems. The data measured on the footrest according to the experiment type are displayed as a contour map in the self-produced software, as shown in Figure 2b.

In the experiment, the data collected from the collection terminal (smartphone) when the rider boards the electric scooter with the data-collection module and footrest were set as one experiment and additional data were repeatedly collected. In addition, the experiments were conducted for two cases depending on the number of riders. In the first case, the rider rides the electric scooter with the foot position collected in two cases: at the center and at the back of the footrest. The experiment was conducted with 10 riders, and 20 data values were collected for each case per rider; a total of 400 data values were collected. The second type is when the driver rides an electric scooter with a passenger. Here, ten riders participated in the experiment, whereby one rider and one passenger were set as a group and alternated to achieve a total of 10 groups. A total of 200 data values were collected by collecting 20 data values for each group.

**Figure 3:**
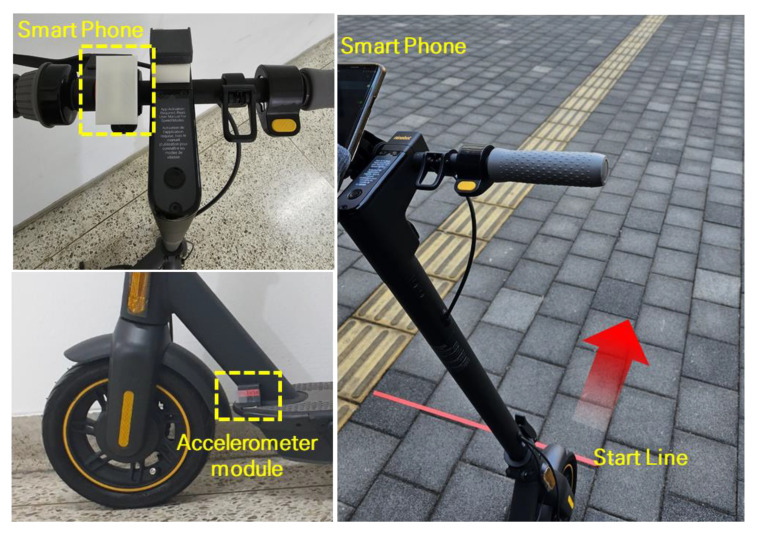
Attachment of the accelerometer module on an electric scooter for the experiment and experimental design for the road-driving data collection.

The manufactured accelerometer module is attached to the electric scooter as shown in Figure 3a. The experimental configuration, as shown in Figure 3b, was used to collect the driving data to prevent collisions with pedestrians or vehicles.

**Figure 4:**
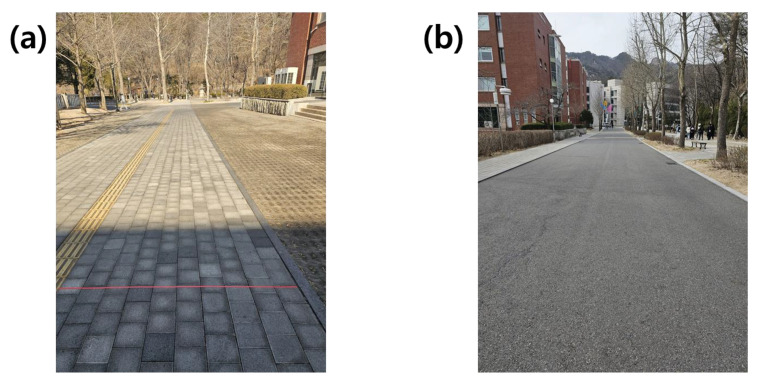
Types of roads driven for the data collection in the experiment: (**a**) sidewalk and (**b**) paved road.

Although electric scooters are not allowed to be driven on sidewalks, most collision accidents with pedestrians occur due to electric scooters driven on sidewalks with the riders unaware of the dangers of this scenario. Such a collision can be prevented by recognizing whether the current location of the electric scooter is a paved road or a sidewalk. In the experiment, the data collection for a certain period of time when the driver was driving an electric scooter with the accelerometer module was set as one experiment, and additional data were collected repeatedly. Data collection was conducted on two types of roads, namely sidewalks and paved roads, as shown in Figure 4. The paved roads used in the experiment were selected as straight roads with minimal incline. The experiment was conducted by collecting the data measured with the accelerometer module while driving at a constant duration and speed.

The electric scooter uses Segway–Ninebot products. The speed of the scooter is expressed in level units on the dashboard, and the exact speed can be determined by connecting the device to the exclusive smartphone application software provided by Segway–Ninebot via Bluetooth. The speed displayed on the dashboard of the electric scooter represents 1 km/h per level. The maximum speed of the electric scooter purchased for research has a speed limit, which was confirmed to be 10 km/h, which is the 10th level. In this experiment, assuming a situation where a collision accident occurred, the driving data were collected for 5 s at a constant speed after reaching the maximum speed of 10 km/h. In this experiment, 400 paved-road-driving data values and 400 sidewalk-driving data values were collected. The data collected from the two types of experiments were analyzed for each type of data, and an AI model was developed according to the dataset. The developed AI model for each type was evaluated for its suitability in problem solving based on performance indicators.

## 3. Results

### 3.1. AI Model Set Up and Evaluation Method

After identifying the characteristics of the data collected in each experiment, a corresponding AI model was designed and trained. Subsequently, the predicted results and performance of the trained AI model and its possibility for solving the problems presented in this study were evaluated. The AI models used in this study used Keras, an AI library, and were developed using Google Colab as the front end and Tensorflow as the back end.

**Figure 5:**
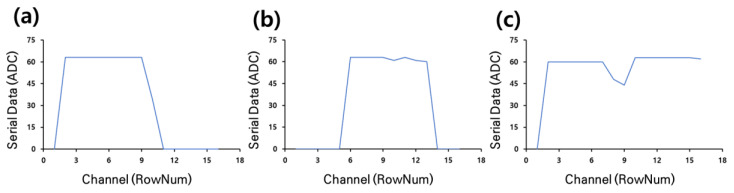
Serial data graph for each number of subjects collected in the experiment according to the coordinates noted in the footrest used for AI learning: (**a**) one person at the back part, (**b**) one person at the middle part, and (**c**) two people boarding.

The first AI model was used to train and test the data obtained using the electric-scooter footrest and data-collection module designed to solve the overloading problem. The boarding data collected through the footrest can be expressed in a graph according to the number and location of the subjects when they board the electric scooter for each type, as shown in Figure 5. The shape of the graph according to each type of experiment has similar output characteristics.

**Figure 6:**
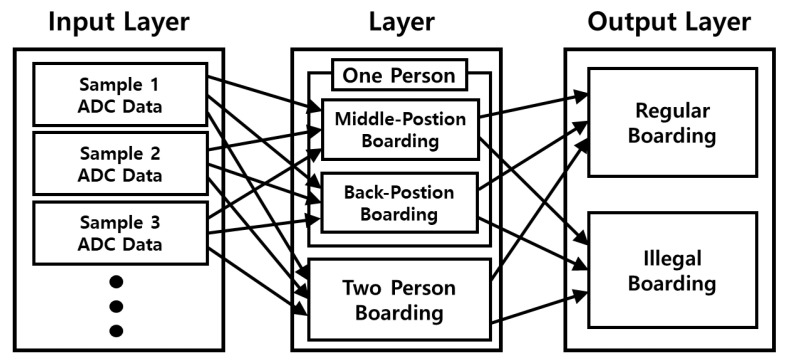
Block diagram for the design of the AI model using the footrest data.

A convolution neural network (CNN), which is a neural network specialized in determining key features in video and time-series data, was used as the AI neural network owing to its characteristics. The AI model developed based on CNN used Softmax, a regularization function, and ReLU, a rectifying linear function, as the activation functions, and adaptive moment estimation (ADAM) based on gradient descent as an optimizer [19]. Figure 6 shows a schematic of the CNN model developed in this study.

**Figure 7:**
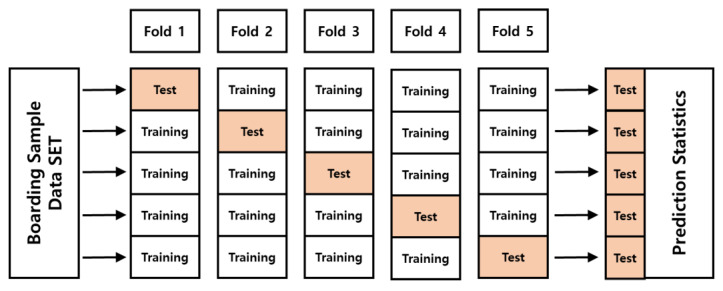
Block diagram of the k-fold cross validation for the CNN-based artificial intelligence model.

We used k-fold cross validation to evaluate the classification performance of the first AI model. K-fold cross validation is a validation method used to ensure reliable accuracy while avoiding overfitting in AI models with relatively small datasets. In particular, it is characterized using all the data as a test set at least once. In this study, five-fold, which is a commonly used value, was applied to the k-value before setting up the k-fold cross validation. The validation method proceeded with the structure shown in Figure 7.

**Figure 8:**
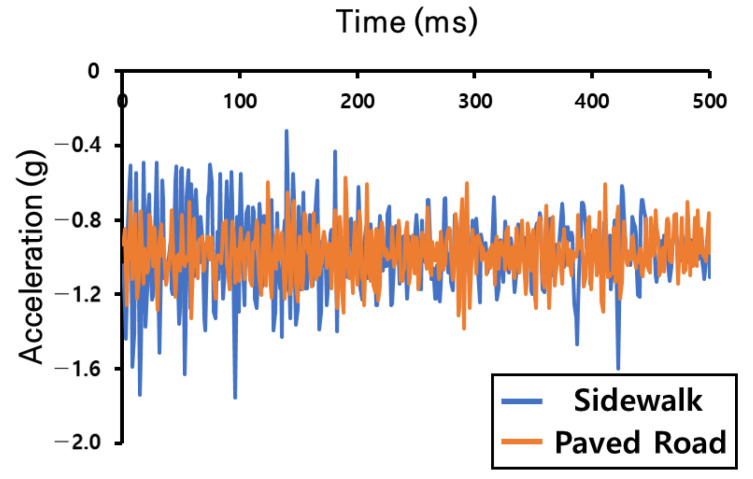
Driving graph of acceleration over time for each road type (paved roads and sidewalks) collected in the experiment used for AI learning.

The second AI model was used to easily train and test the data collected with the accelerometer module, which was designed to solve the problem of contact accidents between pedestrians and electric scooters on sidewalks. The road-driving data collected with the accelerometer sensor module were used as the output graph according to the road type the electric-scooter rider was driving on, as shown in Figure 8.

**Figure 9:**
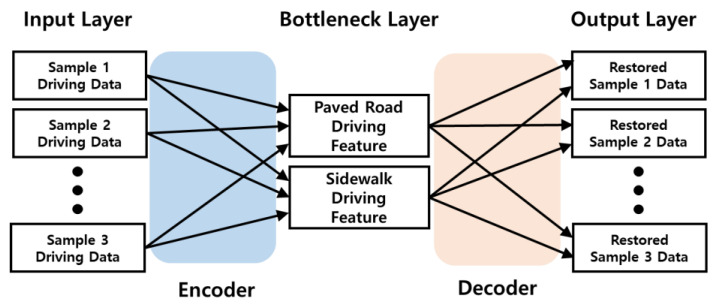
Block diagram for the design of the artificial intelligence model using the road-driving data.

The graph shows the z-axis acceleration values, which are the most variable of the data measured with the accelerometer, graphed over time to show the roughness of different types of roads, taking into account the location of the sensor and the orientation of the scooter. The second AI model used an auto-encoder algorithm, one of the regression analysis models, to identify and classify the causal relationship between the acceleration data over time collected in real time from the accelerometer module and the road type. The auto-encoder algorithm used here implements an anomaly detection method. In particular, it uses training data to build a model that finds objects with characteristics that are different from the existing data and classifies objects with different patterns as anomalies [20]. The AI model developed based on this regression analysis uses LeakyReLU as the activation function and ADAM based on gradient descent as the optimizer. Figure 9 shows the block diagram of the regression analysis model developed in this study.

Loss functions, namely the mean absolute error (MAE) and mean square error (MSE), which are often used as performance evaluation indicators of regression models, were used to evaluate the classification performance of the second AI model. The loss function is an index that expresses the difference between the predicted value of the model and the actual value calculated based on the measured data, thereby indicating the ability of the model to properly represent the data. The MAE can be obtained by averaging the absolute value of the difference between the predicted and actual values. This indicator can easily grasp the degree of learning of the entire data, which is obtained using Equation (1):

(1) $$\mathrm{MAE}=\frac{1}{n}\sum \left|y-\hat{y}\right|.$$

The MSE is the average of the squares of the difference between the predicted and actual values, thereby calculating the error of the correct response rate for the incorrect answer and the error of the correct response rate for the actual correct answer. Its formula is shown in Equation (2).

(2) $$\mathrm{MSE}=\frac{1}{n}\sum {\left(y-\hat{y}\right)}^{2}.$$

where y and $\hat{y}$ are variables common to MAE and MSE, *y* is the actual value of the data used in the model, and $\hat{y}$ is the predicted value of that data.

### 3.2. Evaluation Results

**Figure 10:**
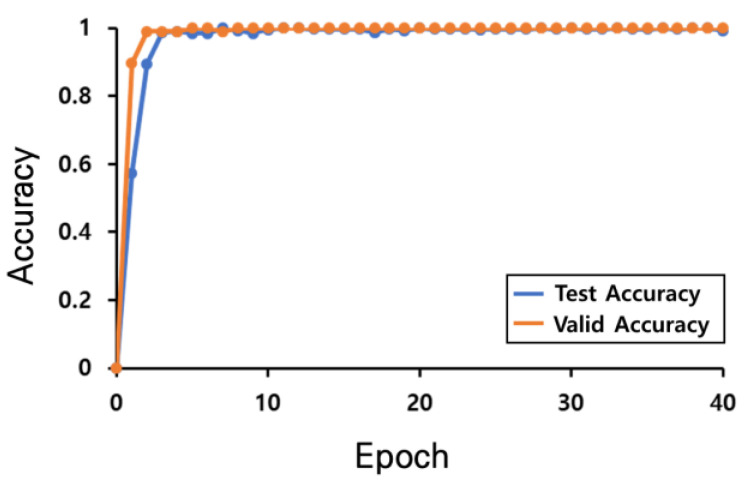
Learning convergence graph using the k-fold validation of the CNN-based AI model using the boarding data.

**Figure 11:**
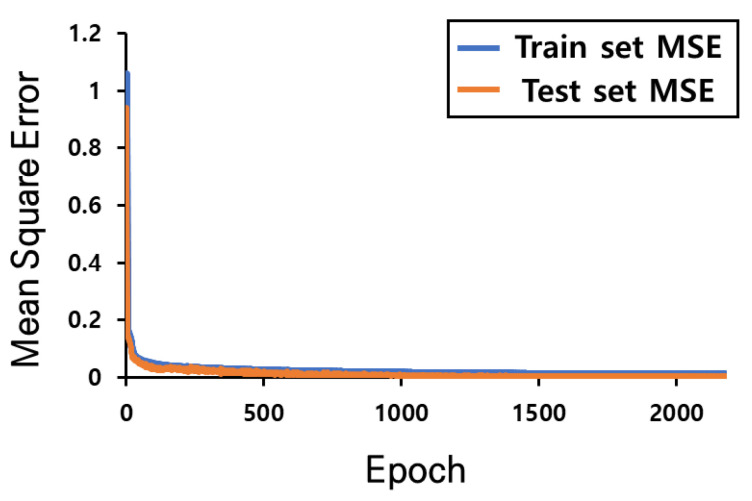
MSE by epoch of the regression AI model using driving data.

**Figure 12:**
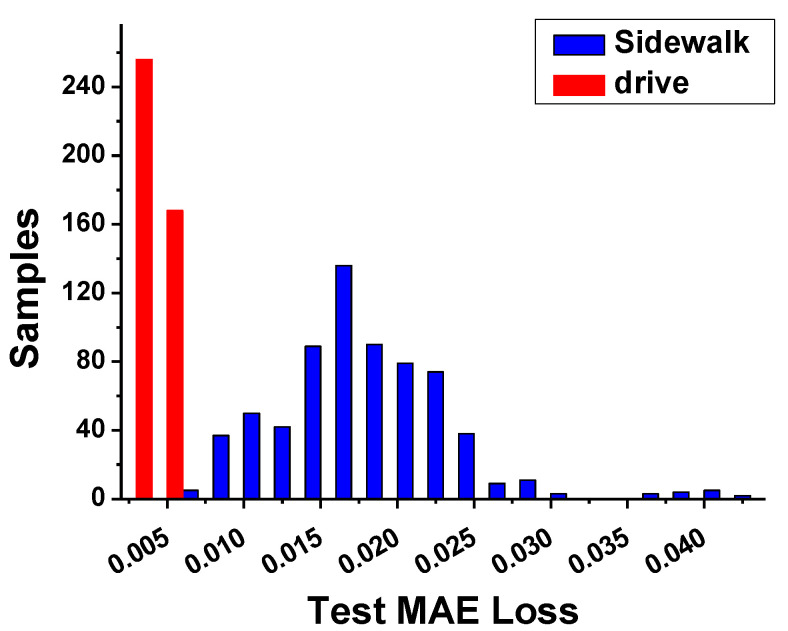
MAE loss graph by road type according to unknown samples in the trained regression AI model.

The learning convergence process using the k-fold verification to verify the CNN-based AI model trained using the boarding data collected in the first experiment is shown in Figure 10. Meanwhile, the learning convergence process of the regression analysis model learned using the driving data collected in the second experiment is shown in Figure 11. The MAE loss is compared by applying 200 unknown road-driving data values and 200 unknown sidewalk-driving data values, which were not used for training the model, to verify the learned AI model; the results are shown in Figure 12.

## 4. Discussion

In this study, an electric-scooter footrest, a data-collection module, and an accelerometer module were developed to prevent various accidents caused related with the rapidly increasing use of electric scooters. The developed module and footrest can be attached to an electric kickboard, and then the rider can receive data from the module by connecting a smartphone when using the electric kickboard. After connecting to the smartphone, various data collected as the rider uses the electric kickboard are transmitted to the artificial intelligence server, allowing the user to check whether multiple people are riding or the electric kickboard is driving on the sidewalk in the current state. The module and collected data are designed as follows. The footrest for the electric scooter was made of a conductive film and was connected to the data-collection module to collect the rider’s boarding data. The data-collection module consisted of an ADC, which received analog signals measured from the footrest and converted them to digital signals, and an Arduino module, which transmitted the collected data. Furthermore, the accelerometer module consisted of an accelerometer that measures the driving data and a Wi-Fi module for wireless communication and transmission of the data transmitted with the data-collection module. In the experiment, the boarding data for different numbers of people boarding an electric scooter and different positions were collected with the footrest and data-collection module. Meanwhile, driving data of the electric scooter on paved roads and sidewalks were collected with the accelerometer module. The AI model trained with the boarding data used CNN, whereby the k-fold verification accuracy converged to 100%. Moreover, another AI model was trained with the road-driving data using a regression model with an auto-encoder. The evaluation of this model demonstrated the MSE converging to zero. Moreover, the MAE loss applied to the test dataset achieved the discrimination between paved roads and sidewalks. Therefore, this study is expected to contribute in reducing the traffic accident rates of rapidly increasing electric scooters by providing a possible solution that can address their related causes.

The AI measurement system developed in this study was not focused on commercialization, so several improvements are needed. First, the AI used in this study is not installed in the module, so it takes time to determine the problem because it must communicate with the AI server to analyze the collected data. Therefore, the time required for the analysis can be shortened using module porting through artificial intelligence firmware development. Second, there is a need to collect and train data under more diverse conditions to avoid overfitting and improve reliability of AI. For example, various conditions include driving situations (rainy, snowy, etc.) and boarding data of passengers with various physical conditions (height, weight, foot size, gender, etc.). Finally, since safety accidents occur due to various factors such as drunk driving and illegal parking in addition to the accident factors mentioned in this study, it is thought to be one way to develop a system that can include and solve these problems. The goal of future research is to address these various improvements to resolve safety issues related to electric scooters through the integration of electric scooters and systems, thereby contributing to the stable settlement of electric scooters, which are recognized as innovative mobility worldwide.

## Data Availability

Please contact the corresponding author for data requests.
